# Double Fixation Technique for Acute Acromioclavicular Joint Dislocation Using the Two-Button Technique and Gracilis Autograft Reinforcement: A Case Report of an Elite Athlete Seeking a Rapid Return to Sports Activity

**DOI:** 10.7759/cureus.62802

**Published:** 2024-06-20

**Authors:** Nikolaos Stefanou, Alexandros E Koskiniotis, Efstathios Konstantinou, George A Komnos, Sokratis Varitimidis

**Affiliations:** 1 Department of Orthopedic Surgery and Musculoskeletal Trauma, University General Hospital of Larissa, Larissa, GRC

**Keywords:** rockwood type iii, professional athlete, tightrope fixation, hamstrings graft, acromioclavicular reconstruction, acromioclavicular repair, acromioclavicular joint/injuries

## Abstract

Acromioclavicular joint dislocation is a relatively common injury in the general population, especially in younger ages. Although acromioclavicular injuries are a well-studied topic, there are many controversies concerning not only the best way to treat them by operative or conservative methods but also determining the most effective fixation methods, providing better functional outcomes, faster rehabilitation protocols, fewer complications, and lower recurrence rates. In this case report, we present a case of type III acromioclavicular dislocation in a young athlete who was treated operatively using a double fixation technique, aiming to address these controversies and offer insights into the optimal management of such injuries.

## Introduction

Acromioclavicular joint dislocation results from a traumatic event involving direct or indirect force, leading to damage to the acromioclavicular joint and a sprain or rupture of the surrounding acromioclavicular and coracoclavicular ligaments that support the joint. Accounting for approximately 9-12% of all shoulder injuries, acromioclavicular dislocations are particularly common in young athletes, occur about five times more frequently in males, and many of these injuries remain underdiagnosed [1,2].

While mild to moderate dislocations often respond well to conservative treatments, there is considerable debate about the optimal approach for more severe injuries, such as type III dislocations according to the Rockwood classification, characterized by more than 100% displacement of the clavicle from the acromion. Untreated or improperly managed type III acromioclavicular dislocations can lead to significant functional impairment and long-term complications [3].

Many procedures have been described for the surgical management of acromioclavicular dislocations; however, none is considered the gold standard. The most suitable surgical approach for grade III acromioclavicular injuries is also a subject of debate in the literature, especially among high-performance athletes and in combat sports where the risk of repetitive injuries is demonstrably higher [4].

In this case report, we present a case of a type III acromioclavicular dislocation in a young athlete treated operatively using a double fixation technique using the TightRope technique and gracilis autograft reinforcement. The main goal of choosing the specific technique was the rapid return of the athlete to full activity and the avoidance of loss of anatomical reduction and functional decline after repeated high-energy injuries.

## Case presentation

**Figure 1:**
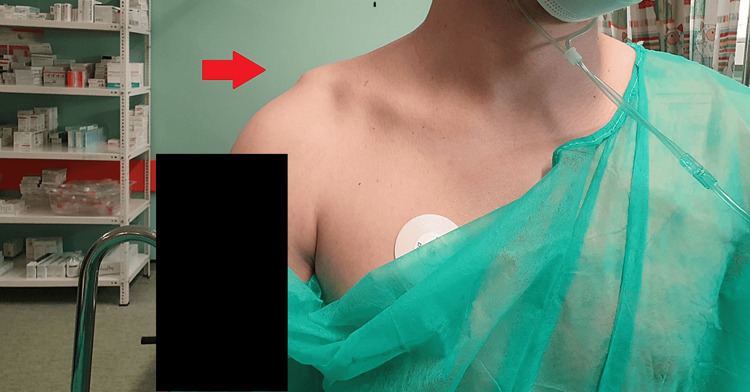
Preoperative clinical image of the injured acromioclavicular joint There is an evident prominence of the dislocated lateral part of the patient’s clavicle.

**Figure 2:**
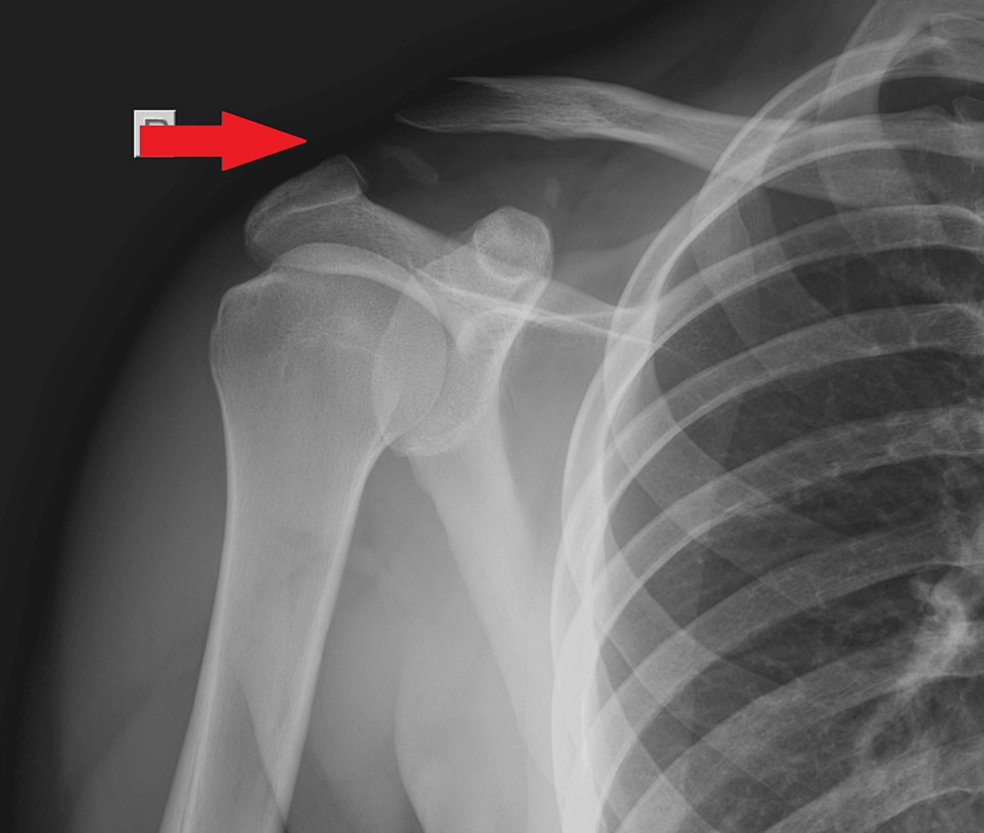
X-ray of a type III acromioclavicular dislocation, accompanied by a small bone fragment from the lateral part of the clavicle

A right-handed 21-year-old male presented to our emergency department with intense pain in his right shoulder following a bicycle fall. Physical examination revealed an evident anterior displacement of the clavicle without skin abrasions (Figure 1), accompanied by a slight crepitus upon palpation of the distal part of the bone. The patient experienced difficulty lifting his arm. Subsequent X-ray examination confirmed a type III acromioclavicular dislocation with a distal clavicle fracture, as per the Rockwood classification (Figure 2).

Given the patient’s status as a professional boxer with high functional demands on his upper limb, it was deemed optimal to admit him to the orthopedic department for surgical intervention.

**Figure 3:**
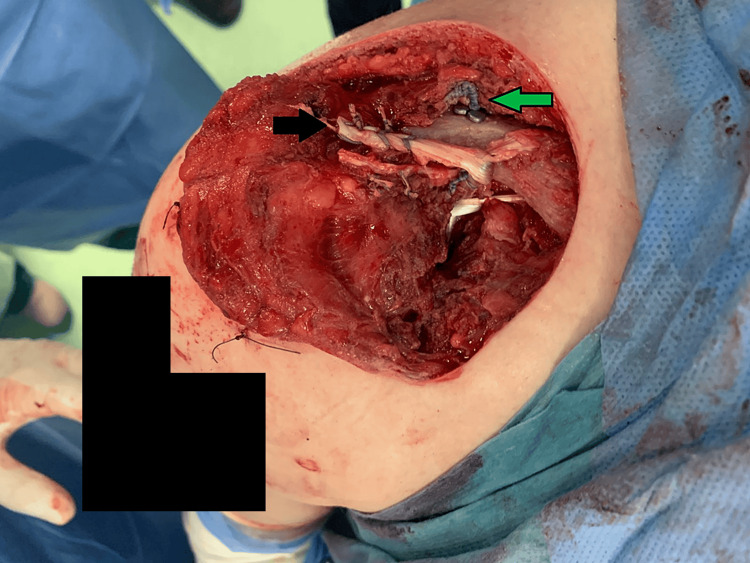
Intraoperative picture of the acromioclavicular joint repair The black arrow indicates gracilis graft, while the green arrow points at the TightRope fixation.

After preoperative interscalene block and induction of general anesthesia, the patient was placed in a beach chair position. The clavicle was stabilized using a combination of two fixation methods. Initially, the distal fragment was excised, and clavicular reduction was achieved by employing the two-button technique with an OrthoCord suture passed through a clavicle and coracoid tunnel (TightRope® fixation, Arthrex, Inc., Naples, United States). A single vertical tunnel is made 3.5-4 cm medially to the lateral edge of the clavicle using a 3.5-mm Dog Bone guide (Arthrex, Inc.) and a 2.4-mm cannulated drill (Figure 3).

**Figure 4:**
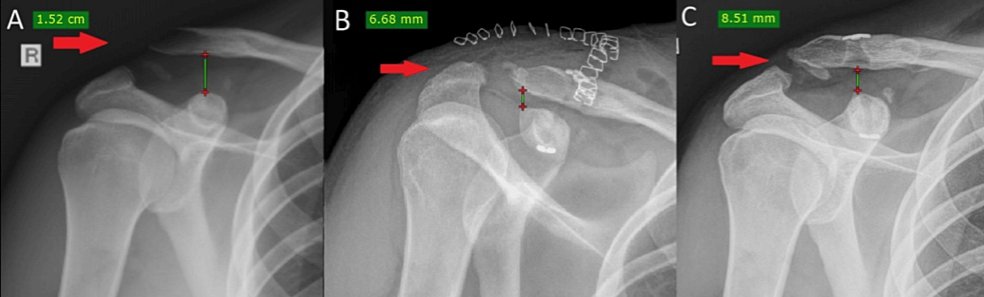
X-ray evaluation of the patient’s injured acromioclavicular joint throughout his follow-up period: (A) preoperative X-ray of the injured shoulder; (B) immediate postoperative X-ray; and (C) X-ray of the injured shoulder one year postoperatively (note that there is no significant loss of the clavicle’s reduction)

The Dog Bone button (Arthrex, Inc.) is introduced through the cannulated drill with the assistance of a suture-passing wire. A Gracilis autograft harvested from the patient’s left knee was employed for acromioclavicular ligament reconstruction, secured in place with sutures. In more detail, a 9-mm- and 2.6-cm-long gracilis tendon autograft was secured in an oval-shaped configuration, around and inferior to the coracoid process and posterior to the medial clavicle. The graft was sutured medial to the cortical button with a No. 2 nonabsorbable suture, while the excess part of the graft was fixed with the same suture type to the posterior portion of the remnants of the acromioclavicular ligament (Figure 3). By this method, both vertical and horizontal stability provided by the coracoclavicular and acromioclavicular ligaments was restored. The immediate postoperative X-ray demonstrated perfect clavicular reduction, which was maintained throughout a follow-up period of one year (Figure 4).

**Figure 5:**
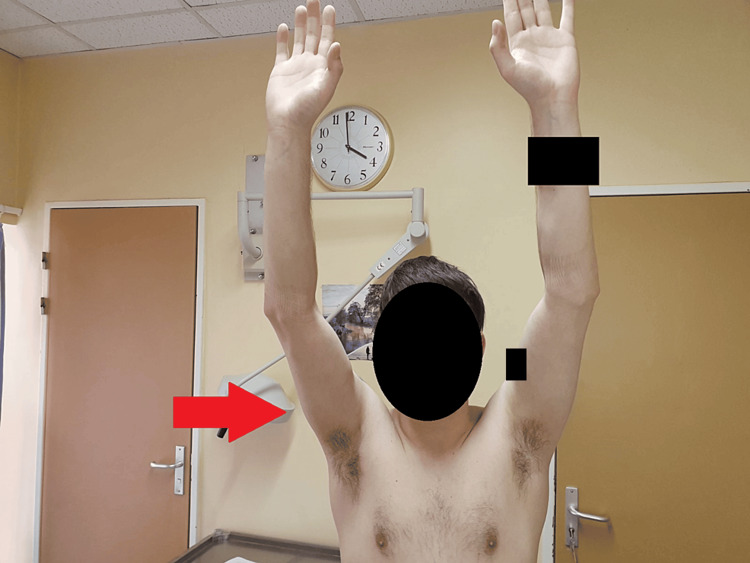
Postoperative clinical outcomes The patient quickly regained full ROM on his injured right shoulder and received clearance to resume sports activities. ROM, range of motion

On the second postoperative day, the patient was discharged with a sling and instructions for range of motion (ROM) elbow exercises and pendulum movement of the shoulder as tolerated. Sling use was discontinued at four weeks; initiating physiotherapy (passive ROM), and active motion below shoulder height strengthening was permitted at six weeks postoperatively. At three months postoperatively, the patient exhibited a full shoulder ROM (Figure 5), an excellent Constant-Murley and QuickDasH score, no recurrent dislocation, and received clearance to resume sports activities.

## Discussion

The management of both acute and chronic acromioclavicular dislocations remains a contentious topic within the orthopedic community [5]. The primary objective of treating acute acromioclavicular joint dislocations is to restore normal joint anatomy and alignment, enabling full power and unrestricted ROM during activity [6]. A systematic review comparing conservative versus surgical management of type III acromioclavicular dislocations, conducted by Longo et al. in 2017, analyzed final outcome measures, recurrence rates postoperation, and complications such as osteoarthritis. The findings revealed no statistically significant differences in these factors between operative and nonoperative treatments, except for a higher incidence of persistent pain in the conservative treatment group. Within the surgical cohort, the re-displacement rate was approximately 14%, with significant variations in recurrence rates among different surgical techniques. Hook-plate, arthroscopic, and MADOCK acromioclavicular procedures demonstrated the lowest recurrence rates, while the Bosworth technique and k-wire fixation exhibited the highest rates. Ultimately, the authors concluded that, although operative methods did not show clear superiority over conservative approaches, there may be potential benefits for patients with high functional demands, such as athletes or heavy laborers [7].

Over the years, numerous surgical techniques have been proposed for the operative management of acromioclavicular type III dislocations, under specific indications. These methods include fixation with a hook-plate, Steinmann pins, wire cerclage, modified Phemister technique, Bosworth screw technique for rigid fixation of the coracoclavicular ligament, or nonrigid fixation with sutures in the Ladermann procedure, utilizing the TightRope system, double-button technique, reinforcement with a semitendinosus autograft tendon, as well as various arthroscopic procedures. While acromioclavicular joint fixation with K-wires and Steinmann pins represented some of the earliest operative treatments for acromioclavicular dislocations, their current utilization is limited due to the potential for severe complications, such as pin migration, and the risk of damage to adjacent neurovascular structures [8]. The hook plate remains one of the most widely used devices for operative treatment worldwide, despite the associated complications. These include the necessity for implant removal after a designated period and the potential for partial loss of reduction as observed post-removal [9]. Nonrigid fixation methods, particularly those employing suture techniques for the coracoclavicular ligaments, have gained popularity in recent years. Studies have shown favorable outcomes compared to older methods such as the Bosworth technique, with lower complication rates and improved functional results and patient-reported outcome measures [10]. This trend is further supported by a systematic review conducted in 2022 by Okereke and Abdelfatah [11].

Recent biomechanical studies have shown that while the coracoclavicular ligaments provide vertical stability, the acromioclavicular ligaments play a crucial role in restraining horizontal forces [12]. Consequently, a combination of stabilization methods for the clavicle in cases of dislocation may yield improved outcomes and lower recurrence rates. Tuxun et al. conducted a study involving 25 patients treated with a clavicular hook plate combined with coracoacromial ligament transposition for acute Rockwood type III, IV, and V dislocations of the acromioclavicular joint. At a mean follow-up of 18 months, 88% of patients reported excellent and good results, according to the UCLA grading system. The mean recovery time for these patients was 12 weeks. The recommended plate removal is around three to four months postoperatively to prevent wear on the acromion and facilitate better healing of traumatized surrounding tissues [13]. To enhance both vertical and horizontal stability, some authors have advocated for a multiple all-suture anchor technique reconstruction of the coracoclavicular ligament, which provides three vertical stabilizers compared to the single stabilizer in the classic TightRope/endobutton technique [14]. Lee et al. demonstrated satisfactory clinical outcomes at a mean follow-up of two years in 27 patients with acute high-grade acromioclavicular joint injury treated with an arthroscopic coracoclavicular fixation technique utilizing multiple soft anchor knots [15]. Saccomanno et al. proposed an alternative operation for reconstructing the acromioclavicular and coracoclavicular ligaments, using one single-strand semitendinosus tendon graft passed underneath the coracoid, through two bone tunnels in the clavicle, and finally looped through an additional tunnel in the acromion. According to the authors, satisfactory clinical and radiological outcomes are expected by their method, with a low failure rate at short-term follow-up [16]. Finally, in 2023, Pérez Rodríguez et al. proposed reconstruction of the acromioclavicular joint using a double augmentation with hamstring tendon and dermal allografts, providing the use of biological supplements in order to replicate the anatomy and functionality of the native ligaments that stabilize the acromioclavicular joint [17].

Due to the lack of unanimity in the choice of the ideal technique for the management of the aforementioned injuries, we propose a novel technique that, in our opinion, ensures anatomical, structural, and biological restoration of the coracoclavicular and posterior-superior acromioclavicular ligaments, providing a stable construction for restoring both horizontal and vertical stability for acromioclavicular type III, IV, and V injuries, according to Rockwood. Its main advantages are summarized in the rapid recovery of the patient, the strength of the construction, the acceptable surgical time, the use of a graft from the patient himself without donor site morbidity, and the combination of static and dynamic restoration of the anatomy. Obviously, it is necessary to evaluate the proposed surgical technique in the future through adequate patient series and comparative studies for both acute and chronic acromioclavicular-coracoclavicular tears.

## Conclusions

Although acromioclavicular joint dislocation is a relatively common injury, it remains a topic of considerable controversy and debate within the medical community. A significant proportion of patients present with acute high-grade acromioclavicular joint injuries, necessitating careful consideration of operative intervention, particularly in light of their activity level and functional demands. Surgical treatment options have advanced significantly in recent years, with a variety of procedures available for addressing acromioclavicular joint dislocations, and new methods continue to emerge. However, there is currently no universally accepted gold standard procedure. Therefore, it is imperative to tailor surgical interventions to individual patient characteristics, including age, physical requirements, and the expertise of the treating surgeon.

## References

[1] Flores DV, Goes PK, Gómez CM, Umpire DF, Pathria MN (2020). Imaging of the acromioclavicular joint: anatomy, function, pathologic features, and treatment. Radiographics.

[2] Cote MP, Wojcik KE, Gomlinski G, Mazzocca AD (2010). Rehabilitation of acromioclavicular joint separations: operative and nonoperative considerations. Clin Sports Med.

[3] Beitzel K, Cote MP, Apostolakos J (2013). Current concepts in the treatment of acromioclavicular joint dislocations. Arthroscopy.

[4] Korsten K, Gunning AC, Leenen LP (2014). Operative or conservative treatment in patients with Rockwood type III acromioclavicular dislocation: a systematic review and update of current literature. Int Orthop.

[5] Maffulli N, Longo UG, Spiezia F, Denaro V (2011). Aetiology and prevention of injuries in elite young athletes. Med Sport Sci.

[6] Bishop JY, Kaeding C (2006). Treatment of the acute traumatic acromioclavicular separation. Sports Med Arthrosc Rev.

[7] Longo UG, Ciuffreda M, Rizzello G, Mannering N, Maffulli N, Denaro V (2017). Surgical versus conservative management of Type III acromioclavicular dislocation: a systematic review. Br Med Bull.

[8] Boffano M, Mortera S, Wafa H, Piana R (2017). The surgical treatment of acromioclavicular joint injuries. EFORT Open Rev.

[9] Kumar N, Sharma V (2015). Hook plate fixation for acute acromioclavicular dislocations without coracoclavicular ligament reconstruction: a functional outcome study in military personnel. Strategies Trauma Limb Reconstr.

[10] Darabos N, Vlahovic I, Gusic N, Darabos A, Bakota B, Miklic D (2015). Is AC tightrope fixation better than bosworth screw fixation for minimally invasive operative treatment of rockwood III AC joint injury?. Injury.

[11] Okereke I, Abdelfatah E (2022). Surgical management of acute rockwood grade III acromioclavicular joint dislocations: a systematic review. Cureus.

[12] Oki S, Matsumura N, Iwamoto W (2012). The function of the acromioclavicular and coracoclavicular ligaments in shoulder motion: a whole-cadaver study. Am J Sports Med.

[13] Tuxun A, Keremu A, Aila P, Abulikemu M, Xie Z, Ababokeli P (2022). Combination of clavicular hook plate with coracoacromial ligament transposition in treatment of acromioclavicular joint dislocation. Orthop Surg.

[14] Jeong JY, Chun YM (2020). Treatment of acute high-grade acromioclavicular joint dislocation. Clin Shoulder Elb.

[15] Lee SJ, Yoo YS, Kim YS (2019). Arthroscopic coracoclavicular fixation using multiple low-profile devices in acute acromioclavicular joint dislocation. Arthroscopy.

[16] Saccomanno MF, Fodale M, Capasso L, Cazzato G, Milano G (2014). Reconstruction of the coracoclavicular and acromioclavicular ligaments with semitendinosus tendon graft: a pilot study. Joints.

[17] Pérez Rodríguez M, Paniagua González A, González Gómez I, Aguado Fernández JP, Minuesa Asensio ÁJ (2023). Reconstruction of the acromioclavicular joint using a double augmentation with hamstrings tendon and dermal graft. Arthrosc Tech.

